# CT findings of *Talaromyces marneffei* infection among HIV patients with lymphadenopathy

**DOI:** 10.3389/fmed.2022.930678

**Published:** 2022-07-27

**Authors:** Xiao-ling Zhu, Guang-Xiao Tang, Xue-yan Liu, Ran Li, Sheng-xiu Lv, Guang-xian Wang

**Affiliations:** ^1^Department of Radiology, Banan People's Hospital, Chongqing Medical University, Chongqing, China; ^2^Department of Radiology, Chongqing Public Health Medical Center, Chongqing, China

**Keywords:** HIV/AIDS, *Talaromyces marneffei*, computed tomography, lymph node, diagnosis

## Abstract

**Background:**

*Talaromyces marneffei* (*T*. *marneffei*) is an opportunistic fungal pathogen commonly found in human immunodeficiency virus (HIV) patients that often infects lymph nodes. Knowledge about the computed tomography (CT) characteristics of *T*. *marneffei* lymphadenopathy in HIV patients is limited. The aim of this study was to investigate the clinical and CT characteristics of *T*. *marneffei* lymphadenopathy to improve its diagnosis and promote recognition of this type of infection in radiology.

**Methods:**

Between February 2019 and June 2021, we retrospectively reviewed the clinical features and CT characteristics of *T*. *marneffei* lymphadenopathy in 21 HIV patients.

**Results:**

The clinical symptoms of *T*. *marneffei* infection are non-specific. Anemia (100%), fever (85.7%) and cough and sputum production (76.2%) were the most frequent symptoms. Multiple lymphadenopathies, mainly in the mediastinum (76.2%) and mesentery (82.4%), can be fused (14.3%) and necrotic (52.4%), with slight (41.7%) and moderate enhancement (58.3%) that is heterogeneous. In addition to involving the lymph nodes, the lesions involved the lungs (81.0%), liver and spleen (42.9%), and small intestine (14.3%).

**Conclusions:**

*T*. *marneffei* is prone to affecting lymph nodes and extranodal organs in HIV patients. Although the clinical manifestations of *T*. *marneffei* infection are not specific, the possibility of *T. marneffei* infection should be considered if CT findings indicate multiple lesion sites.

## Introduction

*Talaromyces marneffei* (*T*. *marneffei*), formerly called *Penicillium marneffei* (*P*. *marneffei*), is an opportunistic infectious fungal pathogen that usually causes disseminated disease and affects multiple organs (1–3). *T*. *marneffei* infection is endemic in tropical regions, especially Southeast Asia, northeastern India and southern China (4). The disease mainly affects immunocompromised individuals, especially those with human immunodeficiency virus (HIV) infection. Given the increasing number of cases of HIV infection, the prevalence of *T*. *marneffei* infection continues to increase (5). Furthermore, *T*. *marneffei* infection is a major cause of HIV-related death, with a mortality rate of up to 30% in this population, even after appropriate antifungal treatment (6).

Most cases of *T*. *marneffei* infection in HIV patients are disseminated in nature, with common presentations including fever, mucosal ulcers, weight loss, anemia, lymphadenopathy, hepatosplenomegaly, respiratory signs, and skin lesions (7–9). However, in clinical practice, because the clinical symptoms are non-specific, the onset of the disease is relatively insidious and mild, and doctors are not familiar with the disease, delayed diagnosis or misdiagnosis often occurs. On the other hand, the diagnosis of *T*. *marneffei* has relied on fungal cultures; however, growth may take up to 4 weeks, and the reported performance has been very variable (7, 9). Hence, rapid diagnosis is of great value to these patients.

*T. marneffei* usually affects multiple organs, such as the skin, lungs, bone, and reticuloendothelial system (10, 11). Radiological examination may be helpful in its diagnosis. However, there are few reports on the radiological findings of *T*. *marneffei* infection. Several studies have reported that the radiological features of *T*. *marneffei* infection in the lungs show multiple exudative shadows with thick-walled cavities, diffuse pulmonary reticular changes, ground-glass opacities, multiple nodules or masses in the bilateral lungs, and bilateral pleural and pericardial effusion (12, 13). Liu et al. (11) showed that bone involvement mainly involved osteolytic bone destruction in flat bones. However, there are few reports on radiological findings of the lymph nodes in HIV *T*. *marneffei*. Hence, in this study, we sought to investigate the radiological characteristics of the lymph nodes in HIV patients with *T*. *marneffei* infection.

## Materials and methods

### Patients

The retrospective study was approved by each participating center's institutional ethics committee, which waived the requirement for informed consent from patients. Between February 2019 and June 2021, the data of 21 HIV patients with *T*. *marneffei* infection and with lymphadenopathy on CT at two participating centers (Banan People's hospital and Chongqing Public Health Medical Center) were retrospectively collected. Patient information, such as sex, age, medical history, auxiliary examination results (e.g., hematological tests, imaging examinations and pathological) and treatment data, was collected from electronic medical records and summarized for analysis by two assessors (Xiao-ling Zhu and Guang-Xiao Tang), who faithfully recorded the clinical data.

### Diagnostic criteria for HIV patients with *T. marneffei* infection

The patients were confirmed to be HIV positive by the Centers for Disease Control, and *T*. *marneffei* infection was confirmed by culture of blood, sputum, skin scrapping, lung, and lymph node samples. While cytology or histopathology was performed using clinical specimens, such as skin lesion, lung, or lymph node samples. A confirmed diagnosed case of *T*. *marneffei* infection met one of following criteria: (1) cultures were characterized by dimorphic fungi that grew as a mold or as a yeast at 25°C or 37°C, respectively; (2) Using cytology or histopathology, tissues or secretions stained with Wright–Giemsa stain or periodic acid-Schiff stain were observed by light microscopy. A transverse septum is a characteristic morphology of the yeast form of *T*. *marneffei* (3). In addition, we perform laboratory tests for CD4 count, peripheral blood test, serum albumin, the concentration of C-reactive protein, and 1-3-β-D glucan to provide an overview of the changes that occur during *T*. *marneffei* infection.

### Imaging protocol and analysis

All patients underwent plain CT, and 12 patients underwent enhanced CT examinations on 64 multidetector CT machines (GE Revolution HD; GE Healthcare, Wisconsin, USA; or Philips Incisive; Philips Medical Systems, Best, Netherlands). All of the CT data were obtained with a section thickness of 0.625 mm or 0.5 mm and reconstructed to generate multiplanar reconstructions (MPRs) and maximum density projections (MIPs).

Two observers (one with 8 years of experience in abdominal imaging and the other with 15 years of experience in radiology diagnostic experience) analyzed the CT images independently. The distribution, size, density, enhancement patterns (rim, homogeneous or mixed enhancement), enhancement degree (low, iso-, or high) and surrounding structure of the enlarged abdominal lymph nodes were analyzed. The enhancement degree of the enlarged lymph nodes was compared with that of the skeletal muscle. The CT numbers were measured by means of same-size region of interest cursors placed on the center of the lymph nodes and on the adjacent skeletal muscle. Low enhancement indicated that the enhancement degree of the lymph node was less than that of the skeletal muscle, isoenhancement indicated that the enhancement degree of the lymph node was close to the skeletal muscle, and high enhancement meant that the enhancement degree of the lymph node was higher than that of the skeletal muscle. A difference of more than 10 Hounsfield units in mean CT number between the lymph node and the skeletal muscle was considered meaningful. Moreover, the presence of concomitant lesions, including lung, the liver, spleen, and intestinal lesions was also recorded. The continuous data were recorded as average values, and any discrepancies in categorical data were re-evaluated by a third reader (who had 25 years of experience in radiology) for subsequent analyses.

## Results

### Clinical manifestations

During the 28-month study period, 28 HIV patients were diagnosed with *T*. *marneffei* infection according to the inclusion criteria. Of these, only 21 patients who had lymphadenopathy and underwent CT were enrolled (18 men and 3 women). The mean age was 43.7±8.85 years for all patients and was 43.5±9.54 years for males and 45.0 ± 0.82 years for females. The clinical characteristics of the patients with *T*. *marneffei* infection with lymphadenopathy are listed in Table 1. Anemia, fever, cough and sputum production, leukoplakia or ulcer of oral mucosa and malaise were the most common symptoms, followed by hepatosplenomegaly, abdominal pain or diarrhea, cutaneous lesions, and dizziness or headaches. One patient had rheumatic heart disease, and the other patients had no comorbidities.

**Table 1:** Demographic information, clinical manifestations, laboratory data and infection sites in HIV patients infected with *Talaromyces marneffei* with lymphadenopathy (*n* = 21).

**Clinical data**	**Patients**	
	**Numbers**	**%**
Male	18	85.7
Anemia	21	100
Fever	18	85.7
Cough and sputum production	16	76.2
Leukoplakia or ulcer of oral mucosa	14	66.7
Malaise	14	66.7
Hepatosplenomegaly	9	42.9
Abdominal pain or diarrhea	6	28.6
Cutaneous lesions	5	23.8
Dizziness or headaches	4	19.1
Laboratory data		
CD4 count	21^*^	100
Hemoglobin concentration	21^*^	100
White blood cell count	2^∧^, 13^*^, 6^N^	9.5, 61.9, 28.6
Serum albumin concentration	20^*^	95.2
C-reactive protein concentration	21^*^	100
1,3-β-D-glucan^#^	13^∧^, 5^N^	72.2, 27.8
Procalcitonin	11^∧^, 10^N^	52.4, 47.6
Infection sites		
Lymph node	21	100
Lung	17	81
Liver and spleen	9	42.9
Small intestine	3	14.3
Skin	5	23.8

* , reduction;

∧ , elevation;

N , normal;

# , 18 patients were tested.

### Laboratory tests

CD4^+^ T cell counts decreased in all patients (100%, mean 48.52 ± 84.11 cells/μL, range 4–375 cells/μL), 20 patients (95.2%) had counts lower than 200 cells/μL, and 12 patients (57.1%) had counts lower than 20 cells/μL. The hemoglobin concentration was decreased in all patients (100%, mean 84.81 ± 16.32 g/L, range 45–120 g/L). The white blood cell counts were increased in two patients (9.5%, 9.65 × 10^9^ cells/L and 13.94 cells/L), decreased in 13 patients (61.9%, mean 2.38 ± 0.44 × 10^9^ cells/L, range 1.68–3.37 × 10^9^ cells/L), and in the normal range in 6 patients (28.6%, range 3.5–9.5 × 10^9^ cells/L). The serum albumin concentration was decreased in 20 patients (95.2%, mean 26.97 ± 3.70 g/L, range 19.5–34.6 g/L). C-reactive protein concentrations were increased in all patients (100%, mean 75.4 ± 63.19 mg/L, range 16.53–230.40 mg/L). 1,3-β-D-glucan levels were increased in 13 patients (61.9%, mean 322.85 ± 289.93 mg/L, range 60–1130.2 pg/mL), 3 patients (14.3%) did not undergo this test, and the levels were in the normal range in 5 patients (23.8%, <60 pg/mL). Serum procalcitonin levels were increased in 11 patients (52.4%, mean 4.52 ± 5.32 mg/L, range 0.56–19.55 ng/mL) and in the normal range in 10 patients (47.6%, range 0–0.5 ng/mL).

### Radiological findings

**Figure 1:**
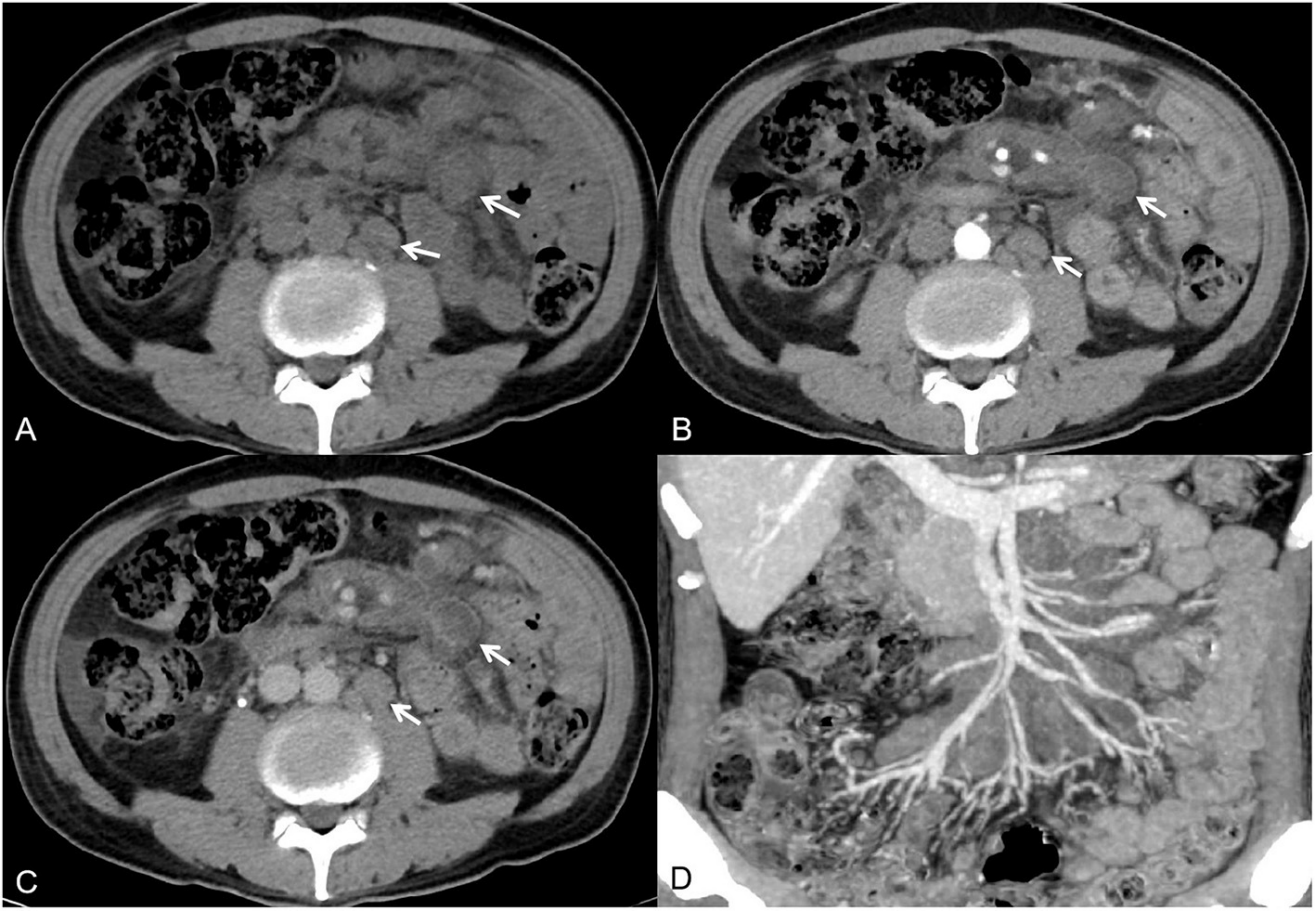
A 54-year-old man had recurrent fever and malaise for 1 month. Axial non-contrast-enhanced CT imaging showed multiple enlarged lymph nodes in the mesentery and retroperitoneum [**(A)**, white arrow]. Axial contrast-enhanced CT images showed lymph nodes with rim or homogeneous low enhancement [**(B,C)**, white arrow]. The maximum intensity projection image showed that the surrounding blood vessels were not invaded **(D)**.

**Figure 2:**
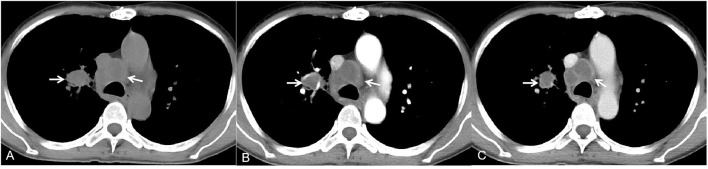
A 59-year-old man with rheumatic heart disease and recurrent cough, sputum and malaise for 3 months. The mediastinal window of the axial non-contrast-enhanced CT image showed multiple enlarged lymph nodes in the right hilus pulmonis and mediastinum [**(A)**, white arrow]. Contrast-enhanced CT images showed lymph nodes with rim low enhancement [**(B,C)**, white arrow].

Of the 21 patients, 17 underwent chest and abdominal CT scans, and 4 underwent chest CT scans only. There were multiple lymphadenopathies observed in the hilus pulmonis (19.0%, 4/21), mediastinum (76.2%, 16/21), mesentery (82.4%, 14/17) and retroperitoneum (76.5%, 13/17). The mean diameter of the enlarged lymph nodes was 15±6.68 mm, ranging from 6 to 33 mm. The shape of lymph nodes was rounded or ovoid, and partial fusion occurred in 3 patients (14.3%, 3/21). The densities of lymph nodes were equal to or slightly lower than the density of skeletal muscle, obvious necrosis was present, and smooth walls were present in necrotic lymph nodes in 11 patients (52.4%, 11/21). Furthermore, these necrotic lymph nodes led to blurred boundaries. The enlarged lymph nodes showed rim enhancement in 2 patients (16.7%, 2/12), homogeneous enhancement in 3 patients (25%, 3/12), and mixed enhancement in 7 patients (58.3%, 7/12). The enlarged lymph nodes showed low enhancement in 5 patients (41.7%, 5/12) and isoenhancement in 7 patients (58.3%, 7/12), and no lymph nodes showed high enhancement. The surrounding tissue and blood vessels were not invaded (Figures 1, 2).

**Figure 3:**
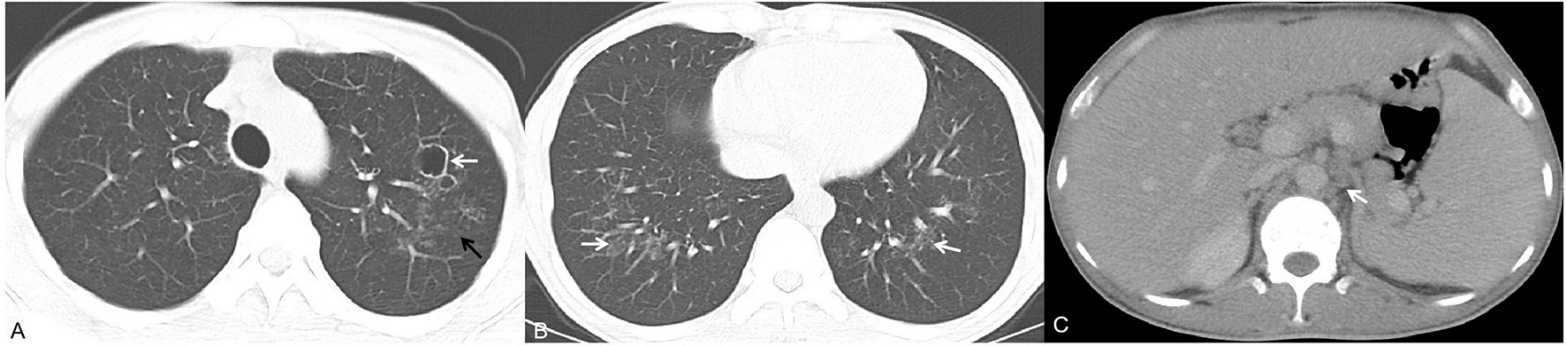
A 40-year-old man presented with fever, cutaneous lesions and ulcers of the oral mucosa. Axial chest CT images **(A,B)** showed multiple cavities, multiple patches (white arrow) and small nodules (black arrow). Moreover, the patient exhibited homogeneous enhancement of hepatosplenomegaly and enlarged lymph nodes in the retroperitoneum [**(C)**, white arrow].

**Figure 4:**
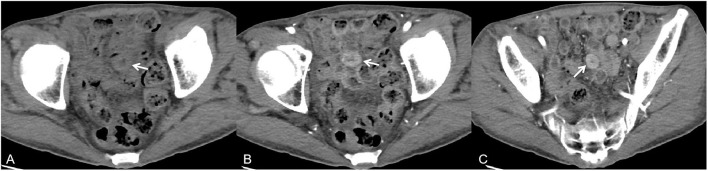
A 57-year-old man presented with cough, sputum, malaise, dizziness or headaches, and abdominal pain for 1 month. Axial non-contrast-enhanced CT imaging showed multiple regions of thickening of the intestinal wall [**(A)**, white arrow]. Axial contrast-enhanced CT images showed lesions with obvious enhancement [**(B,C)**, white arrow].

Beyond the involvement of the lymph nodes observed in patients infected with *T*. *marneffei*, the lesions also involved the lungs in 17 patients, the liver and spleen in 9 patients, and the small intestine in 3 patients. Lung involvement was indicated by multiple patches, massive consolidation, patchy ground-glass opacities, solitary or multiple nodules and masses, small nodules and multiple cavities, and thickening of the interlobular septa and bronchial walls (Figure 3). Liver and spleen involvement was indicated by diffuse enlargement with homogeneous enhancement (Figure 3). Involvement of the small intestine wall was indicated by multiple regions of symmetrical thickening with homogeneous enhancement (Figure 4). Pleural effusion, pericardial effusion and peritoneal effusion were observed in 8, 7, and 7 patients, respectively.

### Fungal culture and histopathology

The diagnosis was confirmed in 20 patients by positive *T*. *marneffei* culture. *T*. *marneffei* was isolated from venous blood (8/21, 38.1%), bone marrow (4/21, 19.0%), sputum samples (3/21, 14.3%), dermal lesion secretions (3/21, 14.3%) and bronchoalveolar lavage fluid (2/21, 9.5%). One patient was diagnosed with *T*. *marneffei* infection by histopathology of lymph node specimens (1/21, 4.8%).

### Treatment and outcome

All patients were treated with various antibiotics before the diagnosis of *T*. *marneffei* infection, but improvements were not observed. After the final diagnosis of *T*. *marneffei* infection, patients were given amphotericin B or voriconazole treatment for 1–4 weeks according to their condition, and itraconazole oral treatment was continued. During the treatment, no deaths occurred, and the clinical symptoms of the patients were significantly relieved.

## Discussion

*T*. *marneffei* infection is rare in patients with normal immunity and commonly occurs in patients with HIV. According to a systematic review by Qin et al. (14), the estimated pooled prevalence of *T*. *marneffei* infection is highest in South China, estimated at 15.0%, while in Chongqing, the prevalence was found to be only 0.2%. Although *T*. *marneffei* infection can be observed in patients without HIV or other non-immunosuppressed conditions, *T*. *marneffei* is considered to be an important opportunistic pathogen associated with HIV (15). As the number of HIV patients increase and the survival time increases, the risk of *T*. *marneffei* infection may increase. Hence, early diagnosis and treatment of *T*. *marneffei* infection may be clinically important.

The clinical symptoms of *T*. *marneffei* infection are non-specific. Limper et al. (16) reported that HIV patients typically exhibited fever, weight loss, hepatosplenomegaly, lymphadenopathy, and respiratory and gastrointestinal abnormalities. Kawila et al. (17) reported that the clinical symptoms of HIV-negative patients with *T*. *marneffei* infections were different from those of HIV-positive patients with *T*. *marneffei* infections. Patients in that study exhibited fever less often and fewer subcutaneous nodules or abscesses. However, two previous studies showed that fever and the presence of subcutaneous nodules were common in HIV-negative patients (3, 13), which is consistent with our research. The reasons for the different clinical manifestations and severity of the disease may depend on the degree of host immunosuppression (4, 15).

Although the diagnosis of *T*. *marneffei* infection can be rapidly made from skin lesions, these lesions are not present in approximately one-third to one-half of patients (2, 18). Laboratory examination can often be used to detect abnormalities in a number of indicators, which can suggest infection, but these methods lack specificity (e.g., white blood cell count and serum albumin concentration): (1) In this study, the decrease in CD4^+^ T cell counts was consistent with HIV infection; (2) The changes of C-reactive protein concentrations and serum procalcitonin level are affected by many factors other than the state of the disease itself; physiological factors and stress reaction can affect the results of C-reactive protein and serum procalcitonin measurements, so these metrics do not have high specificity. In subsequent studies, the patients' disease status could be analyzed in more detail to find correlations from other perspectives; (3) Although HIV patients are more susceptible to fungal infection, the results of the 1,3-β-D-glucan test cannot be used as a diagnostic criterion for *T*. *marneffei* infection. Enlargement of lymph nodes is very common and can result from a variety of diseases, such as infections and malignancies. For superficial lymph nodes, detection and diagnosis can be made by ultrasound, while CT is the recommended radiological examination method for deep-seated lymph nodes.

In this study, the multiple deep-seated lymph nodes, mainly in the mediastinum and mesentery, were found to be fused and necrotic, and mainly with slight and moderate enhancement that was heterogeneous. These CT findings were similar to those previously reported by Li et al. (13), who described 10 HIV-negative patients with *T*. *marneffei* infection and lymph node enlargement. This result suggests that the features of the lymph nodes that are observed in CT are similar, regardless of whether the patient has HIV. However, HIV patients are at high risk of tuberculosis, mycobacterium avium complex (MAC) infection and lymphoma. In clinical practice, *T*. *marneffei* lymphadenopathy may be confused with tuberculosis lymphadenopathy, MAC and lymphoma involving lymph nodes, and differential diagnosis is very important because of the differences in the treatment prescriptions and prognoses between them. Tuberculosis lymphadenopathy usually shows central necrosis with rim enhancement, frequently with a multilocular appearance, and sometimes calcification occurs (19–21). In patients with MAC infection, which almost invariably occurs during the advanced stages of HIV disease, lymph nodes have been found to be characterized by homogeneous soft-tissue attenuation with a clustered pattern (21, 22). Lymphoma typically exhibits homogeneous attenuation with mild contrast enhancement that is more prone to encase the mesenteric fat and vessels, leading to the characteristic cross-sectional “sandwich sign” appearance (21, 23). Moreover, enlarged lymph nodes can also be observed in other diseases, such as metastatic malignancy, leukemia, the persistent generalized lymphadenopathy of HIV infection, cryptococcosis and pyogenic infection. When the location of the primary tumor is identified, most of the metastases can be easily diagnosed (20). Leukemia predominantly presents with homogeneous enhancement of lymph nodes (20). Fine needle aspiration of deep-seated lymph nodes has been reported to enable the diagnosis of *T*. *marneffei* infection rapidly and safely and is beneficial to patients (2).

Hematogenous disseminated *T*. *marneffei* infection is characterized by multiple sites of infection. The CT findings of pulmonary changes in patients with *T*. *marneffei* infection are complicated. Both lungs were found to be affected and showed multiple patches, massive consolidation, patchy ground-glass opacities, solitary or multiple nodules and masses, small nodules, multiple cavities, and thickening of the interlobular septa or bronchial walls, which is consistent with previous research (13). Zhou et al. (24) indicated that liver and spleen enlargement can be observed with focal or multifocal slightly low densities without enhancement. However, in this study, the liver and spleen were involved with homogeneous enhancement. There has been a rare report of small intestine involvement during of *T*. *marneffei* infection (15). CT findings of thickened intestine walls mimic the findings observed with lymphoma, tuberculosis and Crohn's disease (4, 15). Thus, an accurate diagnosis is difficult.

## Limitations

The study has some limitations. First, this was a retrospective analysis with relatively few patients, and a multicenter study with a large sample size is necessary in the future. Second, patients with superficial lymph nodes were not included in this study, and some patients did not undergo abdominal CT and enhancement CT, which may led to selection and results bias. Third, patients with central nervous system symptoms (headaches or dizziness) did not undergo CT or magnetic resonance imaging. Therefore, it remains uncertain whether the central nervous system is involved. Last, all patients were HIV-positive in this study, and the CT characteristics of *T*. *marneffei* lymphadenopathy observed may differ from those observed in HIV-negative patients.

## Conclusions

In conclusion, HIV patients with *T*. *marneffei* infection are prone to exhibiting affected lymph nodes and extranodal organs. Although the clinical manifestations of *T*. *marneffei* infection have no specificity, the possibility of *T. marneffei* infection should be considered if CT findings of multiple site lesions are present. Improving awareness of CT findings, such as lymphadenopathy, and changes in the lung, the liver, spleen and small intestine, has a positive effect on the early diagnosis of the disease.

## Data availability statement

The raw data supporting the conclusions of this article will be made available by the authors, without undue reservation.

## Ethics statement

The studies involving human participants were reviewed and approved by the Medical Ethics Committee of Banan Hospital of Chongqing Medical University. Written informed consent for participation was not required for this study in accordance with the national legislation and the institutional requirements.

## Author contributions

G-xW and S-xL: conceptualization and methodology. X-lZ and G-XT: data curation. X-yL and RL: investigation. X-lZ: writing—original draft. All authors contributed to the article and approved the submitted version.

## Funding

This study was supported by the Joint Project of Science and Health of Chongqing City, China (2022MSXM139).

## Conflict of interest

The authors declare that the research was conducted in the absence of any commercial or financial relationships that could be construed as a potential conflict of interest.

## Publisher's note

All claims expressed in this article are solely those of the authors and do not necessarily represent those of their affiliated organizations, or those of the publisher, the editors and the reviewers. Any product that may be evaluated in this article, or claim that may be made by its manufacturer, is not guaranteed or endorsed by the publisher.
